# Evaluation of time to reimplantation as a risk factor in two-stage revision with static spacers for periprosthetic knee joint infection

**DOI:** 10.1186/s10195-024-00745-7

**Published:** 2024-03-25

**Authors:** Jan Puetzler, Marc Hofschneider, Georg Gosheger, Christoph Theil, Martin Schulze, Jan Schwarze, Raphael Koch, Burkhard Moellenbeck

**Affiliations:** 1 Department of Orthopaedics and Tumor Orthopaedics, Muenster University Hospital, Albert-Schweitzer-Campus 1, 48149 Muenster, Germany; 2 Institute of Biostatistics and Clinical Research, University of Muenster, Schmeddingstraße 56, 48149 Muenster, Germany

**Keywords:** Prosthetic joint infection, Two-stage exchange revision arthroplasty, TKA, THA

## Abstract

**Introduction:**

We investigated the time to reimplantation (TTR) during two-stage revision using static spacers with regard to treatment success and function in patients with chronic periprosthetic joint infection (PJI) of the knee.

**Methods:**

163 patients (median age 72 years, 72 women) who underwent two-stage exchange for chronic knee PJI between 2012 and 2020 were retrospectively analyzed (based on the 2011 Musculoskeletal Infection Society criteria). A cutoff TTR for increased risk of reinfection was identified using the maximally selected log-rank statistic. Infection control, aseptic revisions and overall survival were analyzed using Kaplan–Meier survival estimates. Adjustment for confounding factors—the Charlson Comorbidity Index (CCI) and C-reactive protein (CRP)—was done with a Cox proportional hazards model.

**Results:**

When TTR exceeded 94 days, the adjusted hazard of reinfection was increased 2.8-fold (95% CI 1.4–5.7; *p* = 0.0036). The reinfection-free rate was 67% (95% CI 52-79%) after 2 years and 33% (95% CI 11–57%) after 5 years for a longer TTR compared to 89% (95% CI 81–94%) and 80% (95% CI 69–87%) at 2 and 5 years, respectively, for a shorter TTR. Adjusted overall survival and number of aseptic revisions did not differ between the longer TTR and shorter TTR groups. Maximum knee flexion was 90° (IQR 84–100) for a longer TTR and 95° (IQR 90–100) for a shorter TTR (*p* = 0.0431), with no difference between the groups in Oxford Knee Score. Baseline characteristics were similar (body mass index, age, previous surgeries, microorganisms) for the two groups, except that there was a higher CCI (median 4 vs. 3) and higher CRP (median 3.7 vs 2.6 mg/dl) in the longer TTR group.

**Conclusion:**

A long TTR is sometimes unavoidable in clinical practice, but surgeons should be aware of a potentially higher risk of reinfection.

*Level of evidence*: III, retrospective comparative study.

## Introduction

Periprosthetic joint infection (PJI) is one of the most challenging complications in orthopedic surgery. Complex surgical procedures and lengthy systemic treatments aimed at infection control are a tremendous burden on the affected patients and result in high costs for the health care system [1]. The infection risk after primary total hip or knee arthroplasty is approximately 1–2% [2] but can reach up to 50% in complex, multiply revised cases [3–6].

Two-stage exchange arthroplasty with a temporary polymethyl methacrylate (PMMA) spacer is a common and, in many cases, preferred treatment concept for chronic PJI after total knee arthroplasty (TKA) [7, 8]. The spacer has the task of filling the dead space, stabilizing the joint, maintaining the length of the extremity and releasing local anti-infective substances. Static spacers can bridge large defects but result in temporary arthrodesis. Articulating spacers allow for residual knee motion. While the infection control is similar, knee range of motion and function scores are improved with articulating spacers [9–12].

In clinical practice, surgeons are confronted with the issue of the timing of second-stage reimplantation surgery. While, from the patient’s perspective, a short interval might be preferable to regain mobility, various factors such as comorbidities, clinical examination trends, laboratory results and organizational factors influence the time to reimplantation [13].

A common classification by Trampuz and Zimmerli defines intervals of 2–4 weeks (a short interval) and 6–8 weeks (a long interval) until reimplantation [14]. Other authors suggest 4–6 weeks [15] or 9 weeks between the stages [16]. However, spacer intervals used in published clinical studies range from a few days to several hundred days or even several years, but an interval of around 80–100 days is mostly reported [4, 12, 17–22]. This heterogeneity in clinical practice indicates that the optimal duration of the interval between the stages has not yet been defined [23].

Therefore, this retrospective clinical study evaluated the role of TTR regarding the risk of reinfection, mortality, subsequent aseptic revision surgeries and functional outcome in patients with static knee spacers.

## Methods

After obtaining approval from the local ethics committee (blinded for review), a retrospective query of the institutional electronic database of a single academic tertiary revision arthroplasty and orthopedic oncology department was performed. The study was conducted in accordance with the World Medical Association’s Declaration of Helsinki.

**Fig. 1:**
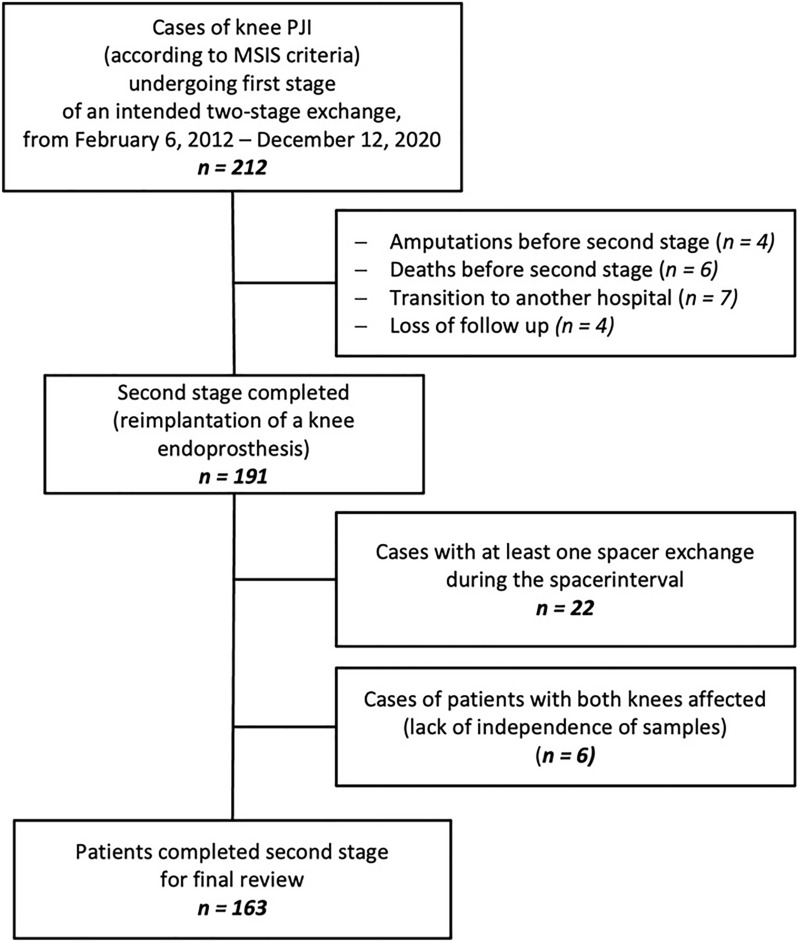
Flowchart of included and excluded cases of knee PJI

We retrieved all 212 cases of chronic knee PJI—diagnosed based on the 2011 Musculoskeletal Infection Society (MSIS) criteria—that underwent the first stage of an intended two-stage exchange between February 6, 2012 and December 12, 2020. We excluded four patients from our analysis who underwent amputation following first-stage surgery and six patients who died after the first stage (Fig. 1). Treatment success and failure were defined as recommended by the MSIS [24]. However, as the TTR was our independent variable, a completed second stage was a precondition for further analysis. Seven patients had a documented transition to another hospital and four patients were lost to follow-up, resulting in 191 cases. Also, 22 cases requiring at least one spacer exchange between the stages were excluded, as this event likely delays the TTR and is considered a risk factor for subsequent treatment failure. Three patients had PJI in both knees (six cases) and were therefore excluded from the analysis, as these cases are not independent. Finally, 163 patients who completed the second stage were included in the analysis.

The electronic patient file was analyzed for patient characteristics, microbiology data and details on the initial surgery as well as potential revision surgeries. The patient’s age, Charlson Comorbidity Index (CCI) [25] and body mass index (BMI) were calculated. The CCI was chosen as a surrogate for host status because it can be well assessed retrospectively from medical records and significantly correlates with the risk of PJI [26, 27].

The diagnosis of infection was made based on the 2011 MSIS criteria [28], relying on culture results, fistula or visible foreign material, as well as synovial leukocyte count and serum C-reactive protein (CRP). In each revision surgery, a minimum of three to five tissue cultures were taken and incubated for a minimum of 7–14 days. All patients underwent a minimum of 6 weeks of combined intravenous and oral tailored antibiotics depending on the microbiological findings. We did not use suppressive antibiotics for planned two-stage revisions to achieve infection control.

A two-stage revision using antibiotic-impregnated polymethylmethacrylate (PMMA) spacers with intramedullary rods was the approach of choice for chronic knee PJI in all patients.

All spacers were handmade using intramedullary titanium rods (diameter: 6 mm) that were wrapped in PMMA to bridge the knee joint and any bone defect present. In our standard protocol, we used a commercially available bone cement (Copal G + C, Heraeus Medical) containing 1 g of clindamycin and 1 g of gentamicin. For every 40-g batch, an additional 2 g of vancomycin was incorporated in the presence of gram-positive organisms or unknown organisms. In instances involving resistant gram-negative microorganisms, we added 2–4 g of meropenem. For fungal infections, 600 mg of voriconazole or amphotericin B was added. For all patients, we recommended that they should not bear weight on the operated leg or, at most, apply light sole contact. Patients who were unable to follow the mobilization with crutches were provided with a wheelchair. We must acknowledge that, in our clinical experience, many patients do not adhere to these instructions, often resulting in larger bone defects due to excessive loading. All patients received a rigid knee brace for additional stabilization.

After completing the minimum of 6 weeks of systemic antibiotics, all patients were seen in the outpatient clinic and were scheduled for reimplantation if the knee joint exhibited no irritation and the laboratory infection parameters (serum CRP and leukocyte counts) were low. Knee joint aspiration was not performed at this stage. All second-stage reimplantation surgeries were performed using a single-design implant system (modular tumor and revision system, MUTARS, Implantcast GmbH, Buxtehude, Germany).

### Description of outcomes: infection control, aseptic revisions, overall survival

Infection control was based on the consensus criteria from Diaz-Ledema, which require healed wounds, no further revision surgery for infection and no PJI-related mortality [29]. Reinfection-free survival was defined as the time from reimplantation (second stage) to the date of the first reinfection of the knee. Patients without reinfection were censored at their last follow-up. Death without prior reinfection was regarded as censored.

Aseptic revisions included all subsequent surgical procedures on the same knee joint that were not performed due to a reinfection. Aseptic-revision-free survival was defined as the time from second-stage revision to the date of aseptic revision. Patients without aseptic revision were censored at their last follow-up or death. Previous reinfections were not considered here.

Overall survival was defined as the time between second-stage revision and death or last follow-up.

### Description of functional outcomes

The functional outcomes were assessed at the last available follow-up at our institution. The Oxford Knee Score (OKS) is a patient-reported outcome (PRO) with 12 questions on activities of daily living. It is considered a highly specific PRO to assess function and pain after TKA [30]. The OKS provides a single summed score which reflects the severity of problems that the respondent has with their knee, ranging from 0 (the worst possible) to 48 (the best outcome). Patients were asked to fill in the German version of the questionnaire on a regular basis during the waiting time for their regular follow-up appointment. These appointments are offered to patients 6 weeks after the second stage if partial weight-bearing has been indicated or after 3 months otherwise. Thereafter, at least annual appointments with a clinical and radiological check-up were scheduled in our outpatient department. In addition, the range of motion of the knee is documented with the neutral-zero method in the electronic patient chart.

### Statistical analysis

Statistical analyses were performed using R statistical software (version 4.2.1, R Foundation for Statistical Computing, Vienna, Austria) and GraphPad Prism (version 9.4.0 for macOS; GraphPad Software, San Diego, CA, USA). All* p* values and confidence limits were two-sided and intended to be exploratory, not confirmatory. Therefore, no adjustment for multiplicity was made. Exploratory two-sided* p* values ≤ 0.05 were considered statistically noticeable.

In descriptive analysis, continuous variables are reported as median (25% quantile–75% quantile, IQR). Absolute and relative frequencies are given for categorical variables. TTR groups were compared using Mann–Whitney* U* tests for continuous data and Fisher’s exact tests for categorical variables.

The optimal cutoff point for the TTR regarding the outcome ‘reinfection’ was identified using the maximally selected log-rank statistic as proposed by Hothorn and Lausen [31] and implemented in the R maxstat package [32].

Time-to-event outcomes were analyzed using log-rank tests and Cox proportional hazard regression models. Event-free rates (at 2 and 5 years) were reported as Kaplan–Meier estimates and pointwise 95% confidence intervals (CI) using log-log transformation. Univariable Cox regressions included either the TTR group or TTR (in days) as the only independent variable. In order to adjust for potential confounding factors, CCI and CRP (mg/dl) were included as additional covariates in the Cox regression. Results are reported as hazard ratios (HR) and corresponding 95% confidence intervals (CI). Median follow-up was estimated using the reverse Kaplan–Meier method.

## Results

### Identification of a TTR threshold

**Fig. 2:**
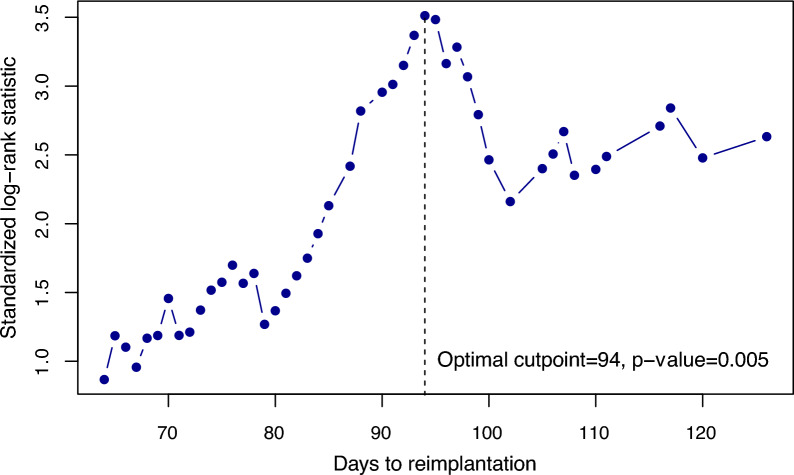
Identification of the optimal cutoff point for the time to reimplantation regarding reinfection-free survival after the second stage in a two-stage exchange arthroplasty of the knee. The optimal cutoff was identified using the maximally selected log-rank statistic as proposed by Hothorn and Lausen [25] and implemented in the R maxstat package [26]

The optimal threshold for TTR regarding reinfection-free survival after the second stage was identified to be 94 days, which was obtained using the maximally selected log-rank statistic (*p* = 0.005) (Fig. 2). Therefore, two groups were created: TTR ≤ 94 days (*n* = 104) and TTR > 94 days (*n* = 59).

### Baseline characteristics

The baseline characteristics of the two groups were similar except for the Charlson Comorbidity Index (CCI) and the C-reactive protein (CRP) level at the first stage (Table 1).

**Table 1:** Baseline characteristics of all patients and the two groups defined according to the time-to-reimplantation cutoff of 94 days

Baseline characteristics	All patients (*n* = 163)	Groups according to time to reimplantation (TTR)		*p *value
		≤ 94 days (*n* = 104)	> 94 days (*n* = 59)	
Age in years, median (IQR)	72 (62–78)	71 (63–77)	72 (61–80)	0.4437*
Sex				
Women,* n* (%)	72 (44%)	47(45%)	25 (42%)	0.7456**
Men,* n* (%)	91 (56%)	57(55%)	34 (58%)	
BMI, median (IQR)	30 (26–35)	30 (26–33)	31 (26–38)	0.2878*
CCI, median (IQR)	3 (2–5)	3 (2–4)	4 (3–6)	0.0056*
Time to reimplantation in days (TTR), median (IQR)	79 (66–110)	70 (62–78)	133 (106–177)	< 0.0001*
Patients with a fistula present at first stage,* n* (%), missing	5 (3%) 28	3 (3%) 14	2 (3%) 14	> 0.9999*
Culture-positive infections,* n* (%)	111 (68%)	71 (68%)	40 (68%)	> 0.9999*
CRP in mg/dl (before first stage), median (IQR), missing	2.8 (1.4–9.2) 6	2.6 (1.0–8.1) 3	3.7 (2.1–14.4) 3	0.0141*
Number of previous aseptic revision surgeries of the same joint, median (IQR), (min–max)	0.0 (0–1) (0–9)	0.0 (0–1.8) (0–5)	0.0 (0–1.0) (0–9)	0.9276*
Number of previous septic revision surgeries of the same joint, median (IQR), (min–max)	0.0 (0–1) (0–9)	0.0 (0–1) (0–9)	0.0 (0–1) (0–6)	0.8059*

Bold font indicates* p* values  ≤ 0.05

^*^ p value from two-sided exact Mann–Whitney* U* test

^**^ p value from Fisher’s exact test

*IQR* interquartile range,* min* minimum,* max* maximum,* BMI* body mass index,* CCI* Charlson Comorbidity Index,* CRP* C-reactive protein

### Treatment outcome: infection control

**Fig. 3:**
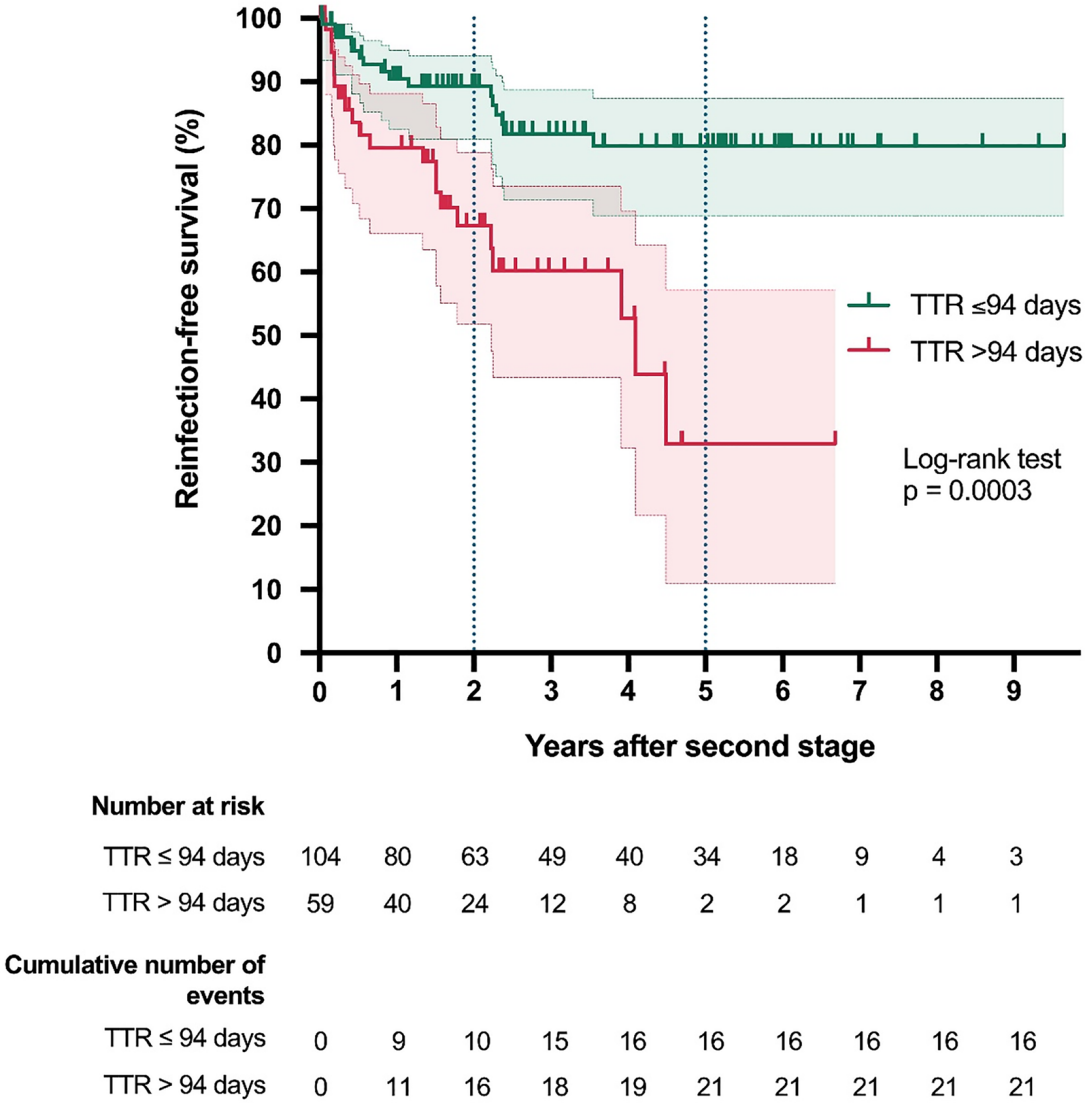
Kaplan–Meier curves of event ‘reinfection’ for the two study groups: time to reimplantation ≤ 94 days in* green* and time to reimplantation > 94 days in* red*. Death without prior reinfection was considered to be censored. The* transparent areas* represent the pointwise 95% CI (log transformed) of the Kaplan–Meier estimates.* Dashed lines* mark the 2-year and 5-year estimates.* TTR* time to reimplantation

In total, reinfection after the second stage was observed in 37 patients, with 16 in the TTR ≤ 94 days group and 21 in the TTR > 94 days group (Table 2). The TTR > 94 days group had a three times higher hazard for reinfection compared to the TTR ≤ 94 days group (HR 3.32, 95% CI 1.71–6.44; *p* = 0.002). The estimated reinfection-free rate in the TTR > 94 days group at 2 years was 67% (95% CI 52–79%) and that at 5 years 33% (95% CI 11–57%), while the corresponding rates in the TTR ≤ 94 days group were 89% (95% CI 81–94%) and 80% (95% CI 69–87%), respectively (Table 2). The whole Kaplan–Meier curves of the two groups differed noticeably (log-rank *p* = 0.0002) (Fig. 3). As the CCI and CRP were noticeably higher at the first stage in the TTR > 94 days group, the Cox proportional hazard regression model for time to reinfection was fitted to adjust for this potential confounder (Table 3, model 1). After adjustment for the baseline CCI and CRP, the hazard for reinfection in the TTR > 94 days group was still increased by a factor of 2.83 compared to the TTR ≤ 94 days group (95% CI 1.40–5.68; *p* = 0.0036).

**Table 2:** Time to event outcomes of the study cohort after the second stage of a two-stage exchange revision for knee periprosthetic joint infection (PJI)†

Treatment outcome	All patients (*n* = 163)	Groups according to time to reimplantation (TTR)		*p* value
		≤ 94 days (*n* = 104)	> 94 days (*n* = 59)	
Follow-up after stage two in months, reverse KM median (95% CI)	38.0 (29.0–50.0)	55.1 (38.0–62.0)	27.0 (22.8–30.5)	0.0002**
**Infection control**				
Number of events (reinfection),* n*	37	16	21	–
HR of reinfection (95% CI)		Reference	3.32 (1.71–6.44)	0.0003*
KM est of reinfection-free rate at 2 years, % (95% CI)	82% (74–87)	89% (81–94)	67% (52–79)	0.0002**
KM est of reinfection-free rate at 5 years, % (95% CI)	68% (58–76)	80% (69–87)	33% (11–57)	
**Aseptic revision surgery after second stage**				
Number of events (aseptic revision),* n*	21	16	5	–
HR of aseptic revision (95% CI)		Reference	0.81 (0.29–2.24)	0.6752*
KM est of aseptic-revision-free rate at 2 years, % (95% CI)	91% (85–95)	88% (79–93)	98% (84–100)	0.6806**
KM est of aseptic-revision-free rate at 5 years, % (95% CI)	76% (64–84)	76% (62–85)	76% (48–90)	
**Overall survival after second stage**				
Number of events (death),* n*	28	17	11	–
HR of death (95% CI)		Reference	2.17 (0.98–4.77)	0.0622*
KM est of overall survival rate at 2 years after stage two, % (95% CI)	89% (82–94)	92% (83–96)	85% (69–93)	0.0494**
KM est of overall survival rate at 5 years after stage two, % (95% CI)	74% (63–82)	80% (68–88)	53% (23–76)	

Bold font indicates* p* values ≤ 0.05

^†^ As the time to reimplantation was the independent variable in our model, only patients who completed stage two were included in the analysis. The patient flow chart in Fig. 1 gives further information on the patients who did not undergo stage two

^*^ p value from the Wald test of the Cox regression including TTR group as the only factor

^**^ Comparison of the whole Kaplan–Meier curves for the TTR≤ 94 days group and  the TTR > 94 days group using the log-rank (Mantel–Cox) test

*HR* hazard ratio,* CI *confidence interval,* KM est *Kaplan–Meier estimate,* reverse KM* reverse Kaplan–Meier method

**Table 3:** Cox proportional hazard regression models for reinfection-free survival and overall survival

	Description	Hazard ratio (95% CI)	*p* value*
**Reinfection-free survival**			
Model 1 (*n* = 157)			
Time to reimplantation	> 94 days vs. ≤ 94 days	2.83 (1.40–5.68)	**0.0036**
CCI	*x* + 1 vs.* x* value	1.12 (0.93–1.35)	0.2250
CRP (at first stage)	*x* + 1 mg/dl vs.* x* mg/dl	1.01 (0.98–1.04)	0.4794
Model 2 (*n* = 163)			
Time to reimplantation (continuous)	*x* + 1 vs.* x* days	1.004 (1.002–1.007)	**0.0068**
	*x* + 7 vs.* x* days	1.03 (1.01–1.05)	–
	*x *+ 30 vs.* x* days	1.14 (1.05–1.23)	–
**Overall survival**			
Model 3 (*n* = 157)			
Time to reimplantation	> 94 days vs. ≤ 94 days	1.92 (0.82—4.51)	0.1321
CCI	*x* + 1 vs.* x* value	1.45 (1.18—1.78)	**0.0004**
CRP (at first stage)	*x* + 1 mg/dl vs.* x* mg/dl	0.98 (0.94—1.02)	0.3530

Bold font indicates *p* values ≤ 0.05

* p values were obtained from the Wald test

*CCI* Charlson Comorbidity Index, *CI* confidence interval, *CRP* serum C-reactive protein

In addition, we examined the effect of TTR as a continuous variable. Therefore, a Cox regression including TTR (days) as the dependent variable was calculated (Table 3, model 2). The hazard for reinfection increased by 14% per additional 30 days of TTR (HR 1.14, 95% CI 1.05–1.23; *p* = 0.0068) and by 3% per additional week of TTR (HR 1.03, 95% CI 1.01–1.05).

### Treatment outcome: aseptic revisions

In total, 21 patients underwent revision for aseptic failure. For all patients, the aseptic-revision-free estimate was 91% (95% CI 85–95%) at 2 years and 76% (95% CI 64–84%) at 5 years, with no noticeable differences between the groups (Table 2).

### Treatment outcome: overall survival

Death was observed in 28 of the patients (TTR ≤ 94 days, * n* = 17; TTR > 94 days, *n* = 11). The overall survival estimate for all patients was 89% (95% CI 82–94%) at 2 years and 74% (95% CI 63–82%) at 5 years (Table 2). Overall survival curves differed slightly between the groups, but the difference was no longer noticeable after adjusting for CCI and CRP (*p* = 0.1321) due to the large influence of CCI on the overall survival (Table 3, model 3).

### Treatment outcomes: functional results

The maximum knee flexion in degrees and the proportion of patients that achieved a knee flexion of > 90° were available in 111 patients (68%) (TTR ≤ 94 days group: *n* = 73 [70%], TTR > 94 days group: *n* = 31 [64%]). The shorter-interval group achieved noticeably higher knee flexion angles compared to the larger-interval group (median: 95° vs. 90°, *p* = 0.0431) (Table 4). Also, a larger proportion of the patients achieved a maximum knee flexion of more than 90° in the shorter-interval group (52% vs. 34%), although the difference was not statistically noticeably different.

**Table 4:** Knee function of all patients and the two groups defined according to the time-to-reimplantation cutoff of 94 days

Knee function at the last follow-up after two-stage exchange was completed	All patients (*n* = 163)	Groups according to time to reimplantation (TTR)		*p* value
		≤ 94 days (*n* = 104)	> 94 days (*n* = 59)	
Maximum knee flexion angle in degrees at last follow-up,* n* (missing), median (IQR)	*n* = 111 (52) 90 (85–100)	*n* = 73 (31) 95° (90–100)	*n* = 38 (21) 90° (84–100)	0.0431*
Patients who achieved > 90° knee flexion at last follow-up,* n* (%) missing	51 (46%) 52	38 (52%) 31	13 (34%) 21	0.1078**
Oxford Knee Score at last follow-up,* n* (missing), median (IQR)	*n* = 79 (84) 17 (11–26)	*n* = 49 (55) 18 (12–30)	*n* = 30 (29) 16 (11–23)	0.2314*

Bold font indicates* p* values  ≤ 0.05

^*^ p value from two-sided exact Mann–Whitney* U* test including TTR group as the only factor

^**^ p value from Fisher’s exact test including TTR group as the only factor

*IQR interquartile range*

Data on the Oxford Knee Score (OKS) were available for 79 patients (49%) (TTR ≤ 94 days group: *n* = 49 [47%], TTR > 94 days group: *n* = 30 [51%]). No noticeable difference was observed between both groups, with a median score of 17 among all patients (Table 4).

### Microbiology

Both groups had similar results regarding the microorganisms detected in intraoperative biopsies at the first stage. The microorganisms were mainly susceptible coagulase-negative staphylococci (*n* = 51, 31%), followed by methicillin-susceptible *Staphylococcus aureus* (*n* = 16, 10%) (Table 5). Cultures were negative at the first stage in 55 patients (34%), and polymicrobial infections were detected in seven patients (4%). At revision surgery for reinfection after a completed two-stage exchange, most of the 37 infections were caused by a minimum of two different species in nine patients (24%), and susceptible coagulase-negative staphylococci were the most common single microorganisms (*n* = 5, 14%). Only four patients with a reinfection (11%) showed a concordance with the initial pathogen identified at first-stage revision, while the microbiological findings did not match the initial findings in 33 patients (89%). There was no noticeable difference regarding the microbiological findings between the two TTR groups.

**Table 5:** Overview of the microorganisms detected in intraoperative biopsies (a minimum of three separate biopsies were performed) of all patients and the two groups defined according to the time-to-reimplantation cutoff of 94 days

Microorganism	All patients	Groups according to time to reimplantation (TTR)	
		≤ 94 days	> 94 days
Microorganism at first stage			
Patients with knee PJI, *n*	163	104	59
CONS, *n* (%)	51 (31%)	33 (32%)	18 (31%)
MRSE, *n* (%)	5 (3%)	2 (2%)	3 (5%)
MSSA, *n* (%)	16 (10%)	11 (11%)	5 (8%)
Gram-negative species, *n* (%)	9 (6%)	7 (7%)	2 (3%)
*Streptococcus *species, *n* (%)	5 (3%)	1 (1%)	4 (7%)
*Enterococcus* species, *n* (%)	5 (3%)	3 (3%)	2 (3%)
Polymicrobial, *n* (%)	7 (4%)	4 (4%)	3 (5%)
Other, *n* (%)	10 (6%)	9 (9%)	1 (2%)
Culture negative, *n* (%)	55 (34%)	34 (33%)	21 (36%)
Microorganism at revision for reinfection			
Patients with a reinfection after two-stage exchange, *n*	37	16	21
CONS, *n* (%)	5 (14%)	1 (5%)	4 (25%)
MRSE, *n* (%)	4 (11%)	4 (20%)	0 (0%)
MSSA, *n* (%)	4 (11%)	2 (10%)	2 (13%)
MRSA, *n* (%)	1 (3%)	0 (0%)	1 (6%)
*Streptococcus* species, *n* (%)	1 (3%)	0 (0%)	1 (6%)
*Enterobacter* species, *n* (%)	1 (3%)	0 (0%)	1 (6%)
*Candida* species, *n* (%)	1 (3%)	0 (0%)	1 (6%)
Polymicrobial, *n* (%)	9 (24%)	3 (15%)	6 (38%)
Other, *n* (%)	2 (5%)	0 (0%)	2 (13%)
Culture negative, *n* (%)	9 (24%)	6 (30%)	3 (19%)

*CONS* coagulase-negative *Staphylococcus*, *MRSE* methicillin-resistant *Staphylococcus epidermidis*,* MSSA* methicillin-susceptible *Staphylococcus aureus*, *MRSA* methicillin-resistent *Staphylococcus aureus*

## Discussion

The time to reimplantation in the concept of two-stage revision for knee PJI was associated with the risk for reinfection in our cohort. If the interval was longer than the calculated threshold of 94 days, patients had a 2.8-fold increase in the hazard of reinfection after adjusting for differences in the Charlson Comorbidity Index and CRP, which were potential confounders.

This result supports the hypothesis from the few existing studies that longer spacer intervals might have a negative influence on infection control. A study by Kubista et al. compared risk factors from 58 patients with reinfections after two-stage exchange of total knee arthroplasty (TKA) with 58 patients they randomly selected from a cohort without reinfection [33]. The median TTR in their study was 66 days in the reinfected group and 61 days in the control group. The hazard ratio for an additional 30 days of TTR was reported to be 1.14 (*p* = 0.03). This value matches well with the hazard ratio of 1.14 that we determined for our cohort for an additional 30 days of TTR. However, Kubista et al. also included a large proportion that required additional revision and spacer exchanges before reimplantation (*n* = 17, 29%). This could be a confounder, as these revisions likely prolonged the TTR and are considered a risk factor for reinfection.

Sabry et al. identified TTR as an independent risk factor among 314 patients with knee PJI who underwent a two-stage exchange, with a median of 124 days until reimplantation in the reinfected group vs. 96 days in the group without reinfection (*p* = 0.015); again, patients requiring a spacer exchange in the spacer interval were not excluded from the analysis [19].

In 2022, Hartman et al. reported on a retrospective cohort of 158 patients with hip and knee PJI who underwent both stages with mainly articulating spacers [34]. The overall reinfection rate was reported as 19.6% (31/158), and the median TTR in the group with reinfection was 141 days compared to 109 days in the group without reinfection, although this difference was not statistically significant (*p* = 0.055). No information on potential revisions between stages was reported.

Recently, Borsinger et al. reported an increased rate of reinfection for the patient group with a TTR of more than 18 weeks after adjusting for comorbidities and the number of previous surgeries [odds ratio, CI 95%: 4.12 (1.18–15.37)] in a cohort of 90 patients with hip and knee PJI (after excluding 11 patients with spacer exchange or Girdlestone resection arthroplasty in the spacer interval) [35]. Two other groups (< 12 weeks and 12–18 weeks) showed no noticeable difference, although the 12–18 weeks group had higher odds of treatment failure [odds ratio, CI 95%: 1.89 (0.67–5.77)]. The arbitrary classification of the groups according to three different TTRs resulted in three groups with similar group sizes. The cutoff values of 12 and 18 weeks that were used were not selected based on the risk of reinfection.

It would be interesting to compare the cutoff calculated with the maximally selected log-rank statistic of their study cohort with our cutoff of 94 days. However, the patient cohort in their study was more heterogeneous than our cohort, as hip and knee PJIs were reported together, and the type of knee spacer was inconsistent (static and mobile, prefabricated and handmade, with some containing polyethylene tibial components in the PMMA).

In a small series by Winkler et al., who prospectively studied a small cohort of 38 patients, reinfection occurred only once during the study period (mean follow-up of 3.3 years) [17]. This shows that even at centers with a high caseload, meaningful results would only be available after a long time. Retrospective clinical studies are therefore necessary to provide a basis for deciding upon TTR using only limited data.

In our cohort, we did not find a relevant difference in the occurrence of subsequent aseptic revisions and mortality after adjusting for confounders.

The average knee flexion ability in the group with a TTR of more than 94 days was lower than that in the group with a shorter interval (90° vs. 95°). This difference may seem small, but 90° of knee flexion is considered a relevant threshold for numerous daily activities [36]. The proportion of patients who achieved more than 90° of flexion was smaller in the group with a TTR of more than 94 days (34% vs. 52%), and the Oxford Knee Score was also slightly reduced, but these differences were not statistically noticeable. Additionally, the small difference between the groups (16 vs. 18) is not considered clinically relevant, as this would require a minimal change of five points [37, 38]. These data were not available for many patients in our cohort, and therefore the lack of statistical validation of the results may be due to the considerably reduced number of patients.

To our knowledge, this is the first study that identified a TTR cutoff based on the risk for reinfection in the largest cohort of knee PJIs that were treated with static spacers. However, several limitations need to be considered when interpreting these results. This is a retrospective analysis that relies on follow-up data, so it is possible that some patients may have undergone revision surgery elsewhere unknown to us. While we assume, considering the complexity of the treatment, that patients will return to our center, the reported data must be considered low-end estimates, and the revision and reinfection burden might be even higher. Due to the retrospective design, this is not a confirmatory but an exploratory approach, and the observed results would need to be confirmed in prospective studies. Although our study reports the CCI as a measure of the overall health status of patients, it is certainly possible that other patient-specific factors that were not recorded, such as soft tissue condition, nutritional status, overall wound healing, or treatment adherence, had a relevant impact on TTR and infection control. These parameters should be taken into consideration for future prospective studies.

In conclusion, this study supports the hypothesis that longer spacer intervals may be associated with reduced infection control after completing two-stage exchange revision. In our cohort of patients with static knee spacers, there was a strong increase in this risk after 94 days.

Therefore, aiming for reimplantation in a two-stage exchange with static spacers for knee PJI before 94 days seems to be reasonable.

## Data Availability

The raw datasets are available from the corresponding author on reasonable request.
